# Metabolic regulation of Th9 cell differentiation: insights for IL-9-driven diseases

**DOI:** 10.3389/fimmu.2025.1672072

**Published:** 2025-09-15

**Authors:** Swetha Peesari, Jeremy P. McAleer

**Affiliations:** Department of Pharmaceutical Sciences, Marshall University School of Pharmacy, Huntington, WV, United States

**Keywords:** Th9 cells, interleukin-9 (IL-9), allergies, autoimmunity, cancer, MTOR activation, PPAR-gamma, ACC1

## Abstract

Th9 cells are a CD4 T cell subset that produces interleukin-9 (IL-9), a pleiotropic cytokine implicated in allergies, autoimmunity and cancer. Defining the cellular effects of IL-9 and factors regulating its expression are essential for fully understanding its roles in immunity and disease. IL-9 acts on a variety of immune and non-immune cells through a heterodimeric receptor composed of IL-9Rα and the common gamma chain. In CD4 T cells, IL-9 promotes mTOR activation, aerobic glycolysis, proliferation and reinforces its own expression. Additional cellular effects include mast cell activation, B cell antibody production and anti-tumor immunity. These biological activities are complemented by recent studies that expand our understanding of Th9 differentiation beyond canonical cytokine and transcription factor pathways. Notably, glycolytic reprogramming and fatty acid metabolism have emerged as key regulators of IL-9 production, mediated through the activities of mTOR, PPAR-γ and acetyl-CoA carboxylase 1 (ACC1). mTOR-driven aerobic glycolysis is essential for Th9 cell differentiation, supporting survival, proliferation, and IL9 expression through HIF-1α activation. In contrast, ACC1 suppresses IL-9 through fatty acid synthesis, which enhances RARα-mediated transcriptional repression. PPAR-γ appears to have dual functions: it promotes IL-9 production by increasing glucose uptake and activating mTOR, but reduces IL-9 in response to synthetic agonists that may increase fatty acid uptake. Overall, these findings highlight critical roles for metabolic regulators in Th9 responses and suggest that targeting these pathways may offer new therapeutic strategies for IL-9-driven diseases.

## Introduction

IL-9 was identified in 1988 as a T helper cell growth factor produced in mitogen-stimulated cultures (1). Originally named P40, IL-9 has 126 amino acids and an unmodified molecular weight of 14 kDa, with post translational glycosylation further increasing the weight to 40kDa (2, 3). Human IL-9 shares 56% amino acid similarity to its murine counterpart (4), with cytokines in both species sharing similar functions. *In vitro*, IL-9 induces the proliferation and activation of IL-4 responsive CD4^+^ helper T cells and mast cells (1, 5), although it may also suppress lymphocyte proliferation under some conditions (6). On the other hand, IL-9 was not observed to stimulate cytotoxic CD8^+^ T cells, B cells or myeloid cells (7). *In vivo*, several inflammatory conditions induce IL-9, including asthma, atopic and contact dermatitis, food allergies and bacterial infections (8–10). These studies and others stimulated interest in identifying cellular sources of IL-9 and the mechanisms regulating its production.

## Cytokine regulation of Th9 cell differentiation

CD4 T cells are the best characterized cell type that produces IL-9, although other cells including mast cells and NKT cells are also capable (11). In 1994, naïve CD4 T cell activation with TGF-β and IL-4 was shown to induce the differentiation of a novel subset later named Th9 (12). Subsequent studies demonstrated indispensable roles for STAT6 and GATA3 in the ability of IL-4 to enhance IL-9 production from T cells treated with TGF-β (13, 14). This was associated with IL-4 suppressing the Treg differentiation factor Foxp3 following TGF-β treatment. On the other hand, IL-10 was expressed at similar levels in murine Th2 and Th9 cells (13). The precise role of GATA3 was not immediately clear: although Th9 cells express negligible levels of the canonical Th2 transcription factor, GATA3 deficiency abrogates IL-9 production. This suggests GATA3 is involved in the early transition of Th2 to Th9 cells prior to its downregulation by TGF-β (13). GATA3 deficiency also increases Foxp3 expression in the presence of TGF-β, highlighting antagonistic functions for these transcription factors. Human Th9 cells share similar differentiation requirements as their murine counterparts, although GATA3 and Foxp3 appear to be expressed at higher levels (15). Other transcription factors involved in Th9 differentiation include PU.1, IRF4, AP1 and NF-kB (7). PU.1 suppresses Th2 cytokines and increases IL-9 production by promoting histone acetylation at the Il9 locus (16). IRF4 contributes to Th9, Th2 and Th17 differentiation (7, 17). Both Th2 and Th17 differentiation conditions can induce IL-9 production from murine CD4 T cells (18). Overall, the coordinated action of multiple transcription factors supports IL-9 production in Th9 cells, which have been classified as a subset of Th2 cells (19). While IL-4 and TGF-β are essential for IL-9 induction, other cytokines have been identified that either increase (e.g. type I IFNs, IL-1β, IL-2, IL-6, IL-9, IL-10, IL-12, IL-21, IL-25) or decrease (e.g. IFN-γ, IL-23, IL-27) IL-9 production from CD4 T cells (7, 14, 15). In addition to Th9 cells, other TGF-β-dependent subsets (e.g. Th17, Tregs) are capable of IL-9 production (18, 20), indicating a degree of functional overlap among these populations.

## Cellular effects of IL-9

IL-9 signals through a heterodimeric receptor composed of IL-9 receptor alpha (IL-9Rα) and the common gamma chain (γc), the latter of which is shared with receptors for IL-2, IL-4, IL-7, IL-15 and IL-21. Thus, IL-9 responsiveness is restricted to cells expressing both subunits, including lymphocyte subsets and mast cells. Inflammatory conditions can broaden IL-9 responsiveness in other populations (e.g. antigen presenting cells and epithelial cells) by upregulating IL-9Rα or γc (21). The binding of IL-9 to its receptor complex primarily activates JAK/STAT signaling pathways, with documented roles for JAK1, JAK3, STAT1, STAT3 and STAT5 (22). Additional signaling pathways include Mitogen-Activated Protein Kinase (MAPK) and insulin receptor substrate (IRS)-Phosphatidylinositol-3 Kinase (PI3K). Negative regulation is mediated by receptor ubiquitination and inhibitory molecules such as SOCS proteins, PIAS and SH-PTP2 (22).

Originally identified as a CD4 T cell growth factor (1), IL-9 induces the expression of genes involved in survival and proliferation. While naïve CD4 T cells lack IL-9R and are non-responsive to this cytokine, several effector subsets are regulated by IL-9. For instance, activating murine CD4 T cells with IL-9 upregulates both IL-4 and TGF-β (18), cytokines integral to the differentiation of Th2, Th9, Th17 and regulatory T cell (Treg) subsets. In Th9 cells, IL-9 acts in an autocrine/paracrine manner to promote effector function (14). This positive feedback loop was attributed to the upregulation of lactate transporter MCT1, leading to aerobic glycolysis (23). In Th2 cells, IL-9 augments IL-5 production (24), while in combination with TGF-β promotes Th17 differentiation (18), possibly due to STAT3 activation by IL-9. The same study also found that IL-9 enhances the suppressive function of Tregs. These findings demonstrate that IL-9 can promote pro- or anti-inflammatory responses depending on the cell type.

**Figure 1:**
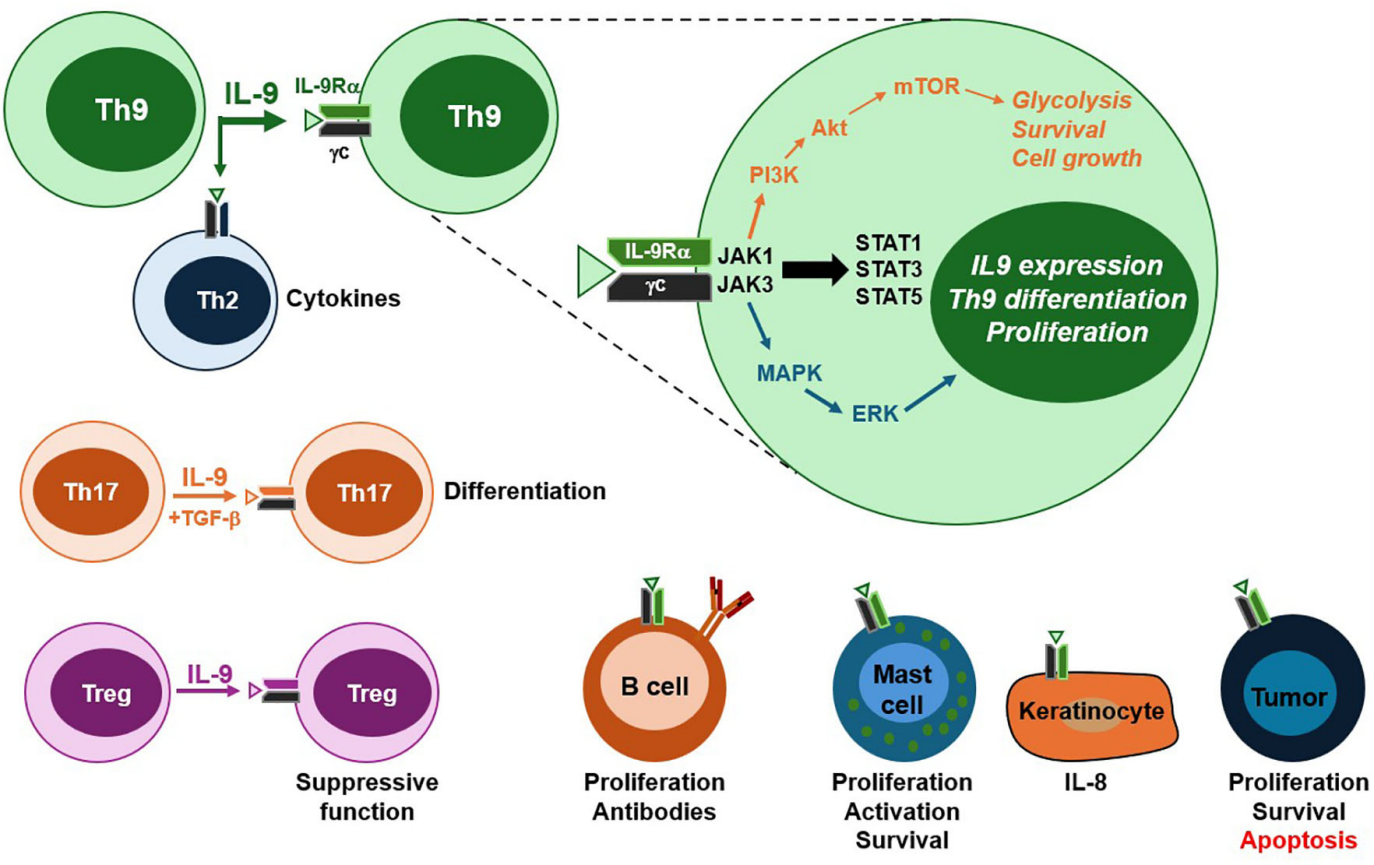
IL-9 receptor signaling and cellular targets. Th9 cells are the primary source of IL-9, although other T cell subsets, including Th17 and Tregs, are also capable of IL-9 production. The IL-9 receptor is a heterodimer composed of IL-9Rα and the common gamma chain (γc). Ligand binding activates three major intracellular signaling pathways. The JAK/STAT pathway promotes Th9 differentiation and IL-9 production. The PI3K/Akt pathway contributes to mTOR activation, resulting in aerobic glycolysis, survival and macromolecule synthesis required for cell growth. The MAP kinase cascade facilitates the transcription of genes involved in effector T cell proliferation. In addition to Th9 cells, IL-9 targets a range of hematopoietic and sometimes non-hematopoietic cells. Among CD4 T cell subsets and B cells, IL-9 enhances proliferation, differentiation and effector function. In mast cells, IL-9 promotes activation, cytokine secretion and survival. Keratinocytes can also respond to IL-9 when they express the receptor complex, leading to pro-inflammatory cytokine production. In cancer, IL-9 plays dual roles through promoting proliferation and survival in lymphomas, while inducing apoptosis in melanoma. The responsiveness of non-hematopoietic populations to IL-9 occurs through the upregulation of IL-9R components in inflammatory settings. Altogether, these pathways and target cell responses contribute to the pleiotropic roles of IL-9 in immunity, inflammation and disease.

Beyond T cells, IL-9 influences several hematopoietic and non-hematopoietic populations. In mast cells, IL-9 enhances proliferation, survival, and activation, leading to increased production of cytokines such as IL-1β, IL-5, IL-6 and IL-13 (5, 11). Transgenic overexpression of IL-9 increases the production of antigen-specific antibodies following immunization, presumably due to IL-9-driven B cell expansion (25). In antigen presenting cells, IL-9 induces TGF-β and suppresses IL-12 (26, 27), dampening Th1 responses. Roles for IL-9 in hematopoiesis have been suggested due to its proliferative effects on IL-3-dependent myeloid cell lines (28). Most non-hematopoietic cells remain non-responsive due to their lack of the γc receptor subunit, although inflammatory conditions may lead to its upregulation. For example, IL-4 enhances IL-9R expression on keratinocytes, and treatment with IL-9 stimulates IL-8 production in an ERK-dependent manner (29). This suggests inflammatory environments can transiently render epithelial cells to be IL-9-responsive. Some tumors also directly respond to IL-9, with protective and pathogenic effects described (30). Lymphoma cell lines show increased proliferation and survival against apoptotic-inducing agents in the presence of IL-9 (31). In contrast, melanoma cell lines upregulate anti-proliferative (p21) or pro-apoptotic (TRAIL) molecules in response to IL-9, enhancing apoptosis (32). Currently, the role of tumor-specific IL-9R expression on Th9-mediated anti-tumor immunity *in vivo* remains unclear. Collectively, the cellular effects of IL-9 support its diverse roles in mucosal host defense, allergic disease, autoimmunity and anti-tumor immunity, as described next (**Figure 1**).

## Role of IL-9 in diseases

IL-9 contributes to inflammatory reactions in the skin, intestine and lungs, as well as cancer and autoimmunity. While not typically the dominant cytokine, IL-9 modulates disease progression through its diverse effects on immune and non-immune populations (21). For instance, atopic dermatitis (AD) is primarily a Th2-driven disease, although Th9 cells are increasingly recognized due to their preferential skin migration (33), elevated IL9/IL9R expression in lesional tissue (19, 34), correlations between Th9 cell frequencies and disease severity (35), and disease amelioration following IL-9 neutralization (36). Mechanisms through which IL-9 contributes to skin inflammation include promoting IgE production, eosinophil and mast cell infiltration, and VEGF secretion from keratinocytes (37). In asthma, IL-9 was identified as a genetic risk factor in 1997 (38), and subsequent studies confirmed its involvement in airway hyperresponsiveness, eosinophilia, mast cell hyperplasia and tissue pathology (17, 39). Ulcerative colitis is another Th2 disease associated with elevated IL-9 expression. Notably, IL-9 deficiency or neutralization protects mice from experimental colitis (40–42). The IL-9-driven intestinal pathology may result from its direct effects on intestinal epithelial cells, leading to decreased growth, proliferation, wound healing and tight junction protein expression (40, 41, 43). Despite its pathogenic role in colitis, IL-9 protects against intestinal nematode infections by enhancing antibody responses, mast cell function and basophilia (44–46). This suggests IL-9 mediated inflammation on epithelial surfaces may have originally evolved as a host defense mechanism.

In cancer, IL-9 plays dual roles acting as either a tumor suppressor or promoter depending on the tumor type (30). Non-hematopoietic models demonstrate potent anti-tumor activities of IL-9 and Th9 cells. For instance, IL-9 directly induces apoptosis and suppresses proliferation in melanoma, enhances CD8 T cell cytotoxicity, and improves mast cell anti-tumor activity (32, 47–49). Notably, endogenous IL-9 inhibits melanoma growth in pre-clinical models, and adoptively-transferred Th9 cells improve survival (47). The anti-melanoma activity is enhanced by stimulation of the Epidermal Growth Factor Receptor (EGFR) during Th9 differentiation (50). Th9 cells themselves may directly contribute to tumor clearance through expression of granzymes and perforin (51). Additional cytokines involved in the anti-tumor activity of Th9 cells include IL-21 and IL-24 (49, 52, 53). In gastric cancer, high IL-9 expression correlates with improved patient survival, and recombinant IL-9 augments the efficacy anti-PD-1 immunotherapy (54). These findings have led to the investigation of Th9 cells in adoptive transfer approaches for solid tumors (30). Conversely, IL-9 can support tumor growth in IL-9R-expressing malignancies, including lymphomas, lung cancer and pancreatic cancer (30, 55–57). IL-9 may also promote immune evasion by upregulating PD-1 on CD8^+^ cytotoxic T cells (58), or enhancing Treg-mediated suppression (59). These dual roles underscore the importance of tumor-intrinsic IL-9R expression and the tumor microenvironment in determining whether IL-9 promotes or inhibits tumor growth. Thus, IL-9 is being explored therapeutically for enhancing its activity in cancer immunotherapy or inhibiting its function in IL-9-responsive malignancies.

Interleukin-9 also has complex roles in autoimmune diseases. Rheumatoid arthritis and psoriatic arthritis patients have elevated IL-9 in synovial fluid and tissues (60, 61). Synovial T cell infiltration was associated with high levels of IL-9R expression, and circulating Th9 cells were expanded by citrullinated peptides. Despite this pro-inflammatory profile, IL-9-deficient mice develop worsened antigen-induced-arthritis, including cartilage destruction and impaired Treg function (62). Similarly, systemic lupus erythematosus patients show elevated IL-9 and Th9 cells in circulation (63, 64), although IL-9 neutralization in lupus-prone mice reduces autoantibody production and renal pathology, especially when combined with IL-17 blockade (65). This was attributed to the ability of IL-9 to promote B cell proliferation and antibody production. In multiple sclerosis models, IL-9 and Th9 cells have been shown to contribute to pathology (66, 67), with IL-9R deficiency either exacerbating or suppressing experimental autoimmune encephalomyelitis (68, 69). These results may reflect IL-9 enhancing both Th17 differentiation and Treg suppressive function (18). Collectively, these findings underscore dual roles for IL-9 in autoimmunity, where it promotes pathology through Th9, Th17 or B cells, while protecting against disease through Tregs and dendritic cells.

## Metabolic regulation of IL-9 production

As with other CD4 T cell subsets, metabolism plays a central role in the differentiation and function of Th9 cells. Several metabolic products, including lipids, amino acids and TCA cycle intermediates, have been shown to directly affect IL-9 production and/or Th9 differentiation (50, 70–73). Here, we highlight roles of the major metabolic regulators mTOR, PPAR-γ and ACC1, drawing upon what is broadly known from CD4 T cell differentiation, as well as recent studies focused specifically on IL-9. Other reviews have also summarized metabolic control of Th9 cells (74, 75).

### mTOR

T cell activation induces profound metabolic changes to support the energy production and biomass accumulation required for clonal expansion and effector cell differentiation. This has been well characterized for glucose metabolism, with Akt and mTOR playing critical roles in driving aerobic glycolysis (76). Among the CD4 T cell subsets, Th9 cells exhibited the highest glycolytic activity, with mTOR required for optimal IL-9 production (77). Accessibility of the IL9 promoter is further enhanced by STAT5-induced histone acetylation, facilitating the binding of transcription factors such as BATF, Foxo1 and HIF-1α (77–80). In the absence of mTOR activation, IL9 transcription is suppressed by Foxp1 and the histone deacetylase SIRT1 (77, 78). Genetic deficiency of SIRT1 increases glycolytic and mTORC1 activity in Th9 cells, enhancing anti-tumor immunity and allergic airway inflammation (77). Importantly, mTOR-driven IL-9 production has been associated with pathology and mast cell hyperplasia in experimental food allergy (81). Both mTORC1 and the rapamycin-insensitive mTORC2 complex contribute to Th9 differentiation, as Rictor deficiency impairs Th9 polarization and allergic airway inflammation (82). Several endogenous molecules can enhance IL-9 production through mTOR. For instance, extracellular ATP induces nitric oxide and mTOR activation, increasing IL-9 production in an HIF-1α-dependent manner (83). Amphiregulin was later shown to activate HIF-1α through the epidermal growth factor receptor (EGFR) (50). The STING ligand 2’3’-cGAMP increases IL-9 in an mTOR-dependent manner, enhancing anti-tumor immunity (84). High glucose concentrations enhance IL-9 production through PPAR-γ mediated aerobic glycolysis (23). This study showed that PPAR-γ suppression decreases mTORC1 phosphorylation, while another demonstrated the microRNA miR-145 suppresses IL-9 through mTOR-HIF1α inhibition (85). Collectively, these studies highlight an indispensable role for mTOR-driven glycolysis in Th9 cell differentiation, and suggest that metabolic interventions targeting this pathway could modulate IL-9-dependent inflammation and immunity.

### PPAR-γ

Peroxisome proliferator-activated receptor-γ (PPAR-γ) is a member of a nuclear receptor superfamily of transcription factors activated by fatty acids (86). Ligand binding drives the expression of genes involved in adipogenesis, lipid metabolism, glucose homeostasis and inflammation (86–88). Clinically, PPAR-γ is targeted by thiazolidinediones for type 2 diabetes and 5-aminosalicylates for inflammatory bowel disease. While its role in adipose tissue insulin sensitivity is well established, distinct functions for PPAR-γ in CD4 T cells have been identified. In response to antigen and costimulation, PPAR-γ promotes fatty acid uptake and lipolysis to support T cell proliferation (89). In Th2 cells, IL-4-induced PPARG expression increases lipid metabolism and IL-5 production (90). *In vivo*, Pparg expression in CD4 T cells contributes to Th2-driven inflammation in the intestine, lungs and skin (91–93). PPAR-γ is also expressed in regulatory T cells (Tregs), where it supports their suppressive function and protects against experimental colitis, psoriasis, graft-versus-host disease and insulin resistance (94–98). Thus, anti-inflammatory effects of PPAR-γ are partially due to the enhancement of Treg-mediated suppression. Interestingly, thiazolidinediones can increase Foxp3 expression and Treg differentiation independently of PPAR-γ (98). Collectively, these studies show that PPAR-γ mediates both pro- and anti-inflammatory functions depending on the T cell subset.

The role of PPAR-γ in Th9 cell differentiation has recently become elucidated. Human Th9 cells were characterized as a subpopulation of Th2 cells expressing high levels of PPAR-γ (19). This population is enriched within memory CD4^+^ CCR4^+^ CCR8^+^ cells in human blood, and the upregulation of IL-9 following T cell activation correlates with a transient decrease in canonical Th2 cytokines IL-4, IL-5 and IL-13. Several genes coordinate the transitioning of Th2 to Th9 cells, including cytokines, growth factors and their receptor signaling pathways (99). PPAR-γ suppression in Th9 cells decreased IL-9 production, as well as the expression of genes promoting T cell activation, glucose metabolism and aerobic glycolysis (19, 23). These pro-glycolytic functions of PPAR-γ occurred under high glucose conditions and were associated with glucose uptake, mTORC1 phosphorylation and proliferation. In line with this, paracrine IL-9 activity increased the expression of genes involved in aerobic glycolysis, including SLC16A1 which encodes for the lactate transporter MCT1, contributing to proliferation (23). The regulatory relationship between PPAR-γ and mTORC1 appears to be bidirectional; however, as another study demonstrated mTORC1 activation prior to PPAR-γ-mediated fatty acid uptake (89). While the endogenous ligand(s) responsible for PPAR-γ activation in Th9 cells remains unknown, the synthetic agonist rosiglitazone suppresses IL-9 in human Th9 cells (100). Combining rosiglitazone with the glucose metabolism inhibitor 2-deoxy-D-glucose decreased IL-9 more potently than either treatment alone, suggesting a glycolysis-independent component of rosiglitazone-mediated suppression. It remains to be determined if this is due to fatty acid uptake or another mechanism, as rosiglitazone exhibits PPAR-γ-dependent and -independent effects on cells (101). Nonetheless, the anti-inflammatory properties of rosiglitazone *in vitro* align with therapeutic benefits of PPAR-γ agonists in IL-9-associated diseases such as atopic dermatitis and psoriasis (91, 102–104). Collectively, PPAR-γ modulators suppress IL-9 production from Th9 cells through metabolic regulation.

### Acetyl-CoA carboxylase 1

T cell activation increases cellular demands for fatty acids, leading to the upregulation of enzymes and transcription factors involved in fatty acid biosynthesis (76, 89). Central to this process is acetyl-CoA Carboxylase 1 (ACC1), which catalyzes the carboxylation of acetyl-CoA to generate malonyl-CoA, the precursor for mid- and long-chain fatty acids (105). In this way, ACC1 supports T cell growth during clonal expansion. Low cellular ATP levels inactivate ACC1 through phosphorylation by AMP-activated protein kinase (AMPK), which diverts metabolism toward fatty acid oxidation to restore ATP levels. Experimentally, ACC1 function can be assessed with pharmacologic inhibitors and/or genetic silencing of its gene ACACA. A mouse model found that diet-induced obesity increases ACC1 expression in memory CD4 T cells, enhancing IL-17 production and Th17-driven pathology (106). Further, ACC1 inhibition reduces IL-17 while increasing Foxp3 expression in Th17 cultures (107), demonstrating its pivotal role in Th17 cell differentiation. Mice lacking ACC1 in CD4 T cells are protected from diseases associated with IL-17, such as asthma, psoriasis and colitis, but show increased susceptibility to infections (108–111). These pro-inflammatory effects of ACC1 are mediated by glycolytic and oxidative metabolic reprogramming (112). Intriguingly, ACC1 inhibition may either enhance or reduce memory CD4 T cell generation depending on the context (113, 114). These studies highlight opposing functions for ACC1 in Th17 and Treg differentiation, raising the question of how this pathway influences other effector subsets such as Th9 cells.

Recent studies identified a suppressive role for ACC1 in Th9 differentiation. Culturing Th9 cells with ACC1 inhibitors or under fatty acid-free conditions substantially increases IL-9 production (100, 115). This increased IL-9 can be restored with exogenous oleic acid or palmitic acid, demonstrating *de novo* fatty acid synthesis and fatty acid uptake suppress Th9 differentiation. At the chromatin level, ACC1 suppression led to greater histone acetylation at the Il9 and Batf3 promoter regions, consistent with a permissive chromatin landscape (115). This may be due to the accumulation intracellular acetyl-CoA following ACC1 suppression, which is used by histone acetyltransferases to globally increase chromatin histone acetylation (116). In addition, ACC1 suppression enhances the sensitivity of cells to TGF-β by increasing SMAD2/3 phosphorylation and its subsequent binding to Il9 (115). The same study demonstrated that retinoic acid receptor-alpha (RARα) signaling may contribute to the suppressive effects of ACC1 and exogenous oleic acid on IL-9 production. This is consistent with RARα activation suppressing Th9 differentiation (117). Pre-treatment of Th9 cells with an ACC1 inhibitor *in vitro* enhanced their anti-tumor activity following adoptive transfer to tumor-bearing mice (115), demonstrating robust Th9 immunity. Human Th9 cultures also contain a small population of Foxp3^+^ IL-9^-^ cells (100), possibly due to exogenous TGF-β or TCR/CD28 stimulation (118, 119). ACC1 suppression increases the ratio of IL-9^+^:Foxp3^-^ cells, demonstrating a pro-inflammatory shift *in vitro* (100). This contrasts with Th17 cultures, in which ACC1 suppression decreases the ratio of IL-17^+^:Foxp3^-^ cells (107). Further studies are necessary to understand differential effects of ACC1 on Foxp3 expression in Th9 versus Th17 cultures. Nonetheless, Foxp3 inhibition did not affect IL-9 production in cultures containing TGF-β (100), suggesting the Foxp3^+^ cells are not functional Tregs. These findings demonstrate that ACC1 limits IL-9 expression through coordinated metabolic and epigenetic mechanisms. Further elucidating how ACC1 limits IL-9 expression will provide key insights into the lipid-mediated control of inflammatory responses.

## Conclusions

**Figure 2:**
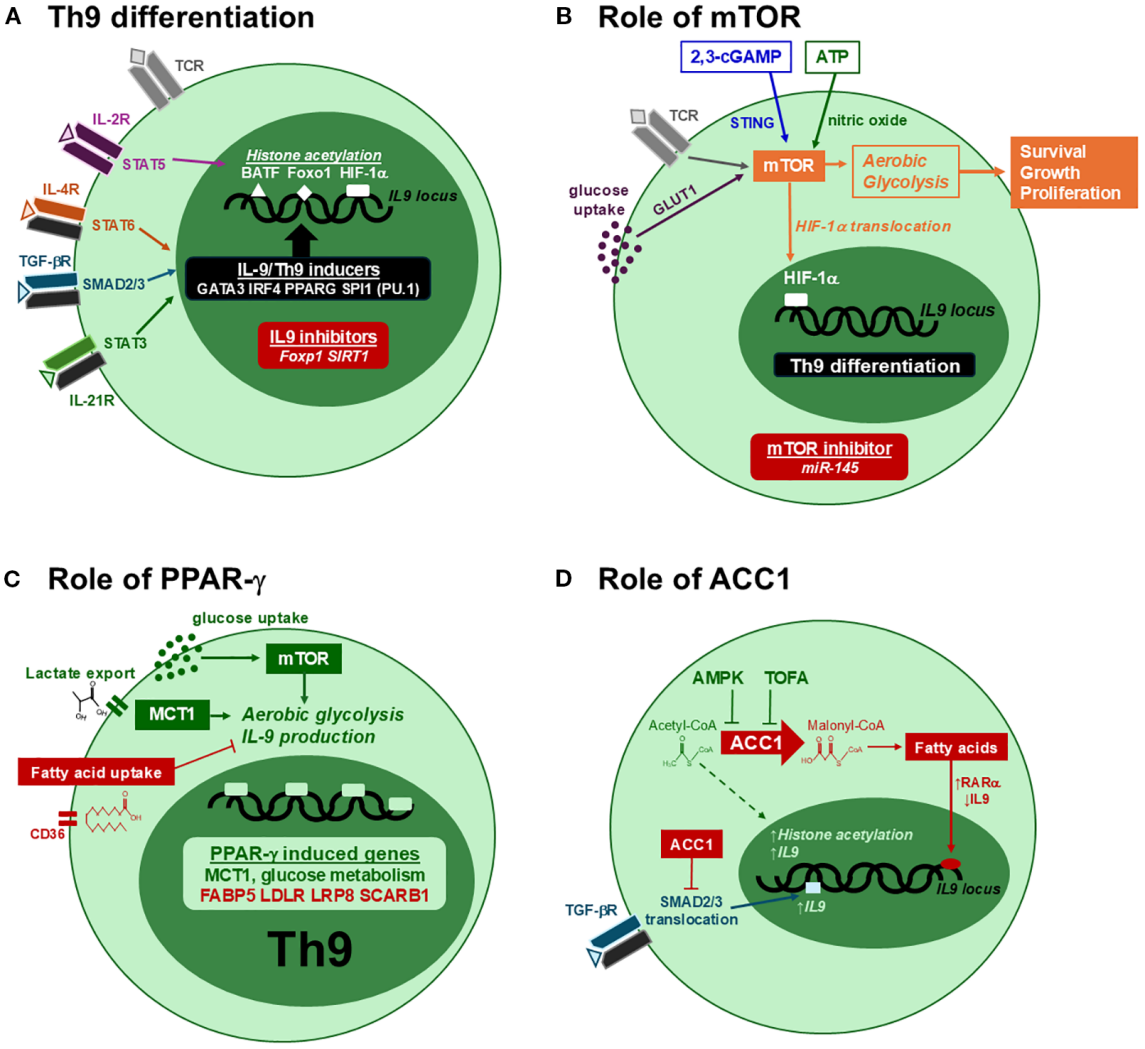
Metabolic regulation of Th9 differentiation. **(A)** Th9 differentiation is induced through the coordinated activities of STAT and SMAD pathways in CD4 T cells. IL-4, TGF-β and IL-21 induce several transcription factors required Th9 differentiation, while STAT5 promotes histone acetylation to enhance their binding to the IL9 promoter. In the absence of Th9-inducing signals, IL9 expression is repressed by Foxp1 and SIRT1. **(B)** mTOR promotes Th9 differentiation through aerobic glycolysis, leading to HIF-1α nuclear translocation, IL9 expression, cell growth and proliferation. This activity is enhanced with high extracellular glucose, 2,3-cGAMP and ATP, and is inhibited by miR-145. **(C)** PPAR-γ has positive and negative effects on IL-9. By enhancing glucose uptake and MCT1-mediated lactate export, PPAR-γ stimulates aerobic glycolysis and IL-9 production. By increasing the expression of genes involved in fatty acid uptake (red), PPAR-γ can also have a negative impact on IL-9. **(D)** ACC1 suppresses IL-9 production through fatty acid synthesis. The conversion of acetyl-CoA to malonyl-CoA generates the substrate for fatty acids, increasing RARα activity and decreasing IL9 expression. In addition, ACC1 suppresses SMAD2/3 phosphorylation in response to TGF-β, further limiting Th9 differentiation. ACC1 suppression increases IL-9 by suppressing fatty acid synthesis and RARα activity, increasing SMAD2 phosphorylation, and possibly promoting histone acetylation via acetyl-CoA (dotted line).

Recent insights into the metabolic regulation of Th9 cells have expanded our understanding beyond canonical cytokine signaling and transcription factor pathways (**Figure 2**). Glycolytic reprogramming and fatty acid metabolism play critical roles in Th9 differentiation and IL-9 production, with important contributions from mTOR, PPAR-γ and ACC1. A more detailed mechanistic understanding will require investigation of additional regulators such as fatty acid synthase (FAS), sterol regulatory element-binding protein 1 (SREBP1) and AMPK. Challenges in using IL-9 targeted therapies may arise from its pleiotropic effects on multiple cell types, including Th9, Th17, Tregs, B cells, epithelial and cancer cells. For instance, the IL-9 neutralizing antibody Medi-528 did not show clinical benefit in an asthma trial (120). In cancer, ACC1 antagonism may facilitate Th9 immunity while also inducing tumor cell apoptosis (100, 115, 121–123). Conversely, ACC1 suppression has been shown to increase Foxp3^+^ Tregs (107), and induce hypertriglyceridemia (124), possibly limiting its therapeutic value. Similarly, PPAR-γ agonists used clinically for metabolic disorders can suppress IL-9 production (100), but may cause adverse effects including weight gain, fluid retention, and bone loss (86). These challenges underscore the need for cell-specific delivery strategies that target Th9 cells or IL-9R-expressing populations. Moving forward, key priorities should include: (A) defining how metabolic pathways influence IL-9-driven diseases, (B) identifying metabolic signatures that distinguish pathogenic from regulatory IL-9R^+^ cell populations, and (C) determining how metabolic interventions can fine-tune IL-9-dependent immunity. As Th9 cells gain recognition for their diverse roles in immune regulation, metabolic targeting may become crucial for harnessing their full therapeutic potential.
